# Health services use among formerly incarcerated Louisiana Medicaid members within one year of release

**DOI:** 10.1371/journal.pone.0285582

**Published:** 2023-05-18

**Authors:** Ashley Wennerstrom, Olivia K. Sugarman, Bruce Reilly, Andrea Armstrong, Angel Whittington, Marcus A. Bachhuber

**Affiliations:** 1 Center for Healthcare Value and Equity, School of Medicine, Louisiana State University Health Sciences Center–New Orleans, New Orleans, LA, United States of America; 2 Department of Behavioral and Community Health Sciences, School of Public Health, Louisiana State University Health Sciences Center–New Orleans, New Orleans, LA, United States of America; 3 Department of Health Policy and Management, Johns Hopkins Bloomberg School of Public Health, Baltimore, MD, United States of America; 4 Voice of the Experienced, New Orleans, LA, United States of America; 5 Loyola College of Law, New Orleans, LA, United States of America; 6 College of Pharmacy, Office of Outcomes Research and Evaluation, University of Louisiana Monroe, Monroe, LA, United States of America; University of Bristol, UNITED KINGDOM

## Abstract

**Objectives:**

To determine the association between enrollment in Medicaid prior to release compared with post-release, and the use of health services and time to the first service use after release among Louisiana Medicaid members within one year of release from Louisiana state corrections custody.

**Methods:**

We conducted a retrospective cohort study linking Louisiana Medicaid and Louisiana state corrections release data. We included individuals ages 19 to 64 years released from state custody between January 1, 2017 and June 30, 2019 and enrolled in Medicaid within 180 days of release. Outcome measures included receipt of general health services (primary care visits, emergency department visits, and hospitalizations), cancer screenings, specialty behavioral health services, and prescription medications. To determine the association between pre-release Medicaid enrollment and time to receipt of health services, multivariable regression models were used which accounted for significant differences in characteristics between the groups.

**Results:**

Overall, 13283 individuals met eligibility criteria and 78.8% (n = 10473) of the population was enrolled in Medicaid pre-release. Compared with those enrolled in Medicaid prior to release, those enrolled post-release were more likely to have an emergency department visit (59.6% versus 57.5%, p = 0.04) and hospitalization (17.9% versus 15.9%, p = 0.01) and less likely to receive outpatient mental health services (12.3% versus 15.2%, p<0.001) and prescription drugs. Compared with those enrolled in Medicaid prior to release, those enrolled post-release had a significantly longer time to receiving many services including a primary care visit (adjusted mean difference: 42.2 days [95% CI: 37.9 to 46.5; p<0.001]), outpatient mental health services (42.8 days [95% CI: 31.3 to 54.4; p<0.001]), outpatient substance use disorder service (20.6 days [95% CI: 2.0 to 39.2; p = 0.03]), and medication for opioid use disorder (40.4 days [95% CI: 23.7 to 57.1; p<0.001]) as well as inhaled bronchodilators and corticosteroids (63.8 days [95% CI: 49.3 to 78.3, p<0.001]), antipsychotics (62.9 days [95% CI: 50.8 to 75.1; p<0.001]), antihypertensives (60.5 days [95% CI: 50.7 to 70.3; p<0.001]), and antidepressants (52.3 days [95% CI: 44.1 to 60.5; p<0.001]).

**Conclusion:**

Compared with Medicaid enrollment post-release, pre-release Medicaid enrollment was associated with higher proportions of, and faster access to, a wide variety of health services. Regardless of enrollment status, we found prolonged times between release and receipt of time-sensitive behavioral health services and prescription medications.

## Introduction

The United States is the world leader in incarceration, with 1.2 million people, or 449 per 100,000 adults, behind bars in 2021 [1]. Most people in custody are released at some point, with almost 444,000 people exiting state or federal prisons in 2021 alone [2]. The transition from long-term incarceration to the community is a critical period that poses major health challenges and an elevated risk of mortality for returning individuals [2–4]. Navigating re-entry can be complex, as people with a history of incarceration confront significant challenges including housing insecurity and homelessness [5], higher rates of unemployment than the general population [6], limited educational attainment [7], and high rates of limited health literacy [8]. Formerly incarcerated people also have higher rates of chronic and infectious disease than the general population [2, 9, 10] and face disparities in access to care [11].

One strategy to increase access to care post-release is to facilitate early enrollment in Medicaid prior to release. The expansion of Medicaid under the Affordable Care Act ensured that most people returning from prison are eligible for health care coverage [12]. While the federal Medicaid Inmate Exclusion Policy [13] precludes coverage for people in custody, except in cases of hospitalization for at least 24 hours, many states have adopted strategies to facilitate enrollment for those leaving incarceration [14, 15]. For example, a total of 43 states suspend Medicaid benefits during incarceration rather than terminating enrollment to facilitate rapid reactivation of benefits post-release [16]. Some states that suspend coverage, such as Louisiana, also have programs to enroll individuals or reactivate their Medicaid benefits prior the end of incarceration, with benefits becoming active immediately upon release [17].

Louisiana’s policy environment regarding health and incarceration is worthy of study. It is the only state in the Deep South (i.e., plantation states including Louisiana, Mississippi, Alabama, Georgia, and South Carolina that exploited the labor of enslaved people prior to the Civil War) to expand Medicaid [18], and has one of the nation’s highest rates of incarceration, at 568 per 100,000 adults, with a total prison population of roughly 26,000 as of 2021 [1]. Due to issues of prison overcrowding, Louisiana houses approximately half of people in state custody in dozens of local jails [19] (usually reserved for those awaiting trial or sentencing or serving a sentence of less than one year). These jails may lack sufficient staff and facilities to offer health care for those with longer sentences. There is evidence that people in Louisiana state custody have limited access to health care services, including preventive cancer screenings, and that formerly incarcerated people face substantial barriers to accessing care and medication after release [20–23].

In January 2017 the Louisiana Department of Health and the Louisiana Department of Public Safety and Corrections (DPSC) created the Justice Involved Pre-release Program (Program) post-Medicaid Expansion to facilitate Medicaid enrollment and coverage for people nearing the end of their sentence and newly eligible under Expansion [18]. The Program is described in detail elsewhere [18]. Briefly, DPSC administrative staff identify people nearing release from incarceration, present them with a comparison of benefits offered by the state’s five contracted Medicaid managed care organizations, and complete enrollment paperwork. Those who are identified as having high medical or behavioral health needs (e.g. HIV, serious mental illness, substance use disorder, multiple medical issues) may choose to participate in case management services offered by the managed care organization they select as their insurance provider. Individuals may opt out of Medicaid enrollment prior to release, and some may not be offered the opportunity to do so if they are released unexpectedly (e.g., if they are paroled of have their conviction overturned). Those who do not enroll prior to release may do so later or not at all.

A recent evaluation of the Program found that it was initially successful in increasing Medicaid enrollment [24]. While gaining access to health coverage after contact with the criminal legal system may not be sufficient to ensure use of some health services [25], there is some evidence that Medicaid coverage upon release facilitates use of health care [24, 26], including among people with a history of substance use [27] or serious mental illness [28]. With limited exception [29], there is a paucity of information about whether the timing of enrollment (i.e. pre-or post-release) affects health services use and time to service use among Medicaid members returning from incarceration. An improved understanding of whether pre-release Medicaid enrollment facilitates access to health services has implications for expanding pre-release Medicaid enrollment programs and improving coordination of care for people leaving incarceration.

The purpose of this study was to examine the impact of Medicaid enrollment prior to release compared to post-release on the use of health services and time to service use among formerly incarcerated Louisiana Medicaid members within one year of release from state custody. The aims were to 1) determine population characteristics, Medicaid enrollment status, health services used, and time to receiving health services after release, and 2) examine the association between enrollment in Medicaid pre-release versus post-release and health service use, including time to health service receipt. We hypothesized that people enrolled in Medicaid prior to release would be more likely to receive health services and have shorter times to accessing services.

## Methods

### Overview

We conducted a retrospective cohort study of Louisiana Medicaid claims data crosslinked with Louisiana Department of Public Safety and Corrections data. To create the dataset, all available Louisiana Medicaid claims data were matched with all available Louisiana Department of Public Safety and Corrections data by social security number, as in a previous analysis [24]. Data were fully anonymized prior to analysis.

### Study population

Inclusion criteria were: 1) age 19 to 64 at the time of release, corresponding with Medicaid Expansion eligibility criteria; 2) release from Louisiana state custody between January 1, 2017 and June 30, 2019; 3) enrollment in Louisiana Medicaid within 180 days of release from state custody; and 4) a valid social security number present in both Louisiana Medicaid claims and Louisiana Department of Public Safety and Corrections release data. Exclusion criteria included dual Medicare-Medicaid eligibility and release out of state, to another carceral facility or type of custody, or due to death. If someone was released more than once, the most recent release was selected to reflect the most up-to-date health service claims available. For people who died during the study period after release, data was kept and analyzed only for services they received prior to death. Of note, Louisiana Department of Public Safety and Corrections houses roughly half of people in state custody in local jails rather than state prisons [19]. Thus, the study population of people released from state custody includes people who were housed in state prisons and local jails.

### Health conditions and service use

We extracted data on health services used by the study population including general and behavioral health service visits, cancer screening, and filling of prescription drugs within 365 days of release. We selected these health services to represent a broadly applicable mix of medical and specialty behavioral health services relevant to and prioritized by formerly incarcerated individuals, according to prior research [20–23]. General health services included primary care, emergency department visits, and inpatient hospitalization. Specialty behavioral health services included psychiatrist visits, specialized outpatient mental health services (other than psychiatry), inpatient mental health services, specialized substance use disorder outpatient services, specialized substance use disorder residential services, and medication for opioid use disorder. Medication for opioid use disorder included buprenorphine and extended-release injectable naltrexone, as well as methadone treatment, which became covered by Louisiana Medicaid as of January 20, 2020. Cancer screening included cervical cancer, colorectal cancer, and breast cancer screenings, which were selected because eligibility for screening could be reasonably estimated with data available (i.e., age and sex). We aligned our definition of those eligible for cervical cancer screening (female, age 21 to 64), colorectal cancer screening (age 50 and older), and breast cancer screening (female, age 50 and older) with United States Preventive Services Task Force recommendations during the study period and included all recommended methods (e.g., colonoscopy, fecal immunochemical tests, fecal occult blood tests, and all others recommended for colon cancer screening) in our analyses. Prescription drugs included antihypertensives, insulin and hypoglycemics, inhaled bronchodilators and corticosteroids, HIV antiretrovirals, contraceptives, and cancer chemotherapy as well as antidepressants, antipsychotics, and mood stabilizers. We additionally attempted to analyze the use of hemodialysis, hysterectomy, and hernia repair but had to remove these analyses due to very small sample sizes. We calculated time to service use as the number of days between the release date and the first date of service receipt. If a person used a service more than once, only the first service relative to release date was used.

### Medicaid enrollment

We identified individuals who were enrolled in Medicaid prior to release by examining Medicaid enrollment files and date of release. As Medicaid enrollment is on a calendar month basis, we considered pre-release enrollment as a member who was enrolled during or prior to the same calendar month as release from state custody.

### Other characteristics

We extracted sociodemographics including age at release, sex, race/ethnicity, and education level. For race/ethnicity, we recoded American Indian/Alaska Native and Asian individuals to the missing, other, or unknown category due to small cell sizes. We also extracted incarceration information including length of incarceration, the release site type (i.e., prison or jail), and the calendar year of release.

### Statistical analysis

First, using a complete case analysis, we calculated descriptive statistics to examine characteristics and health service for the overall study population and by Medicaid enrollment pre-release versus post-release. Next, we compared characteristics and health service use by Medicaid enrollment pre-release versus post-release using Mann-Whitney U tests for continuous variables and Chi square tests for categorical variables. To determine the association between Medicaid enrollment pre-release versus post-release and time to health service receipt, we used generalized linear models which accounted for clustering at the level of the release facility. We used time to receipt of a given health service as the outcome variable and adjusted for all characteristics that were significantly different between the two groups (sex, length of incarceration, release from jail or prison, calendar year of release). We did not include race/ethnicity in the models due to the large amount of individuals with a missing or unknown value. All models used a gamma distribution with a log link, which was selected based on the distribution of the variables. We used Stata version 15.1 for all statistical analyses.

This study was approved by the Institutional Review Boards at the Louisiana State University Health Sciences Center–New Orleans and the Louisiana Department of Health. Informed consent was not required, as this was a retrospective cohort study. Results of this study were reported according to the STrengthening the Reporting of OBservational studies in Epidemiology (STROBE) guidelines.

## Results

Of 13287 individuals meeting study criteria, complete data were available for 13283. Most individuals were male (83.0%, n = 11020), of missing, other, or unknown race/ethnicity (38.8%, n = 5159), had a 12^th^ grade or high school equivalent education (42.6%, n = 5655), and were released from a local jail (70.2%, n = 9330; Table 1). The median age was 36.3 (interquartile range [IQR]: 29.4–45.1) and the median length of incarceration was 0.9 years (IQR: 0.4–2.3). Overall, 78.8% (n = 10473) were enrolled in Medicaid prior to release.

**Table 1:** Characteristics of Louisiana Medicaid members released from state custody between January 1, 2017 and June 30, 2019.

Characteristic	Overall study population (n = 13283)^a^	Enrolled in Medicaid pre-release (n = 10473)	Enrolled in Medicaid post-release (n = 2810)	*P*
Age, median (IQR)	36.3 (29.4–45.1)	36.3 (29.4–45.3)	36.3 (29.5–44.3)	0.37
Female sex, n (%)	2263 (17.0)	1941 (18.5)	322 (11.5)	<0.001
Race/ethnicity, n (%)				<0.001
Black	4113 (31.0)	3215 (30.7)	898 (32.0)	
White	3657 (27.5)	2882 (27.5)	775 (27.6)	
Hispanic/Latino, of any race	354 (2.7)	242 (2.3)	112 (4.0)	
Missing, other, or unknown	5159 (38.8)	4134 (39.5)	1025 (36.5)	
Education level, n (%)				0.62
Less than high school	1139 (8.6)	907 (8.7)	232 (8.3)	
Some high school	4676 (35.2)	3667 (35.0)	1009 (35.9)	
12^th^ grade or high school equivalent	5655 (42.6)	4452 (42.5)	1203 (42.8)	
Some college	1205 (9.1)	955 (9.1)	250 (8.9)	
No educational information	608 (4.6)	492 (4.7)	116 (4.1)	
Years of incarceration, median (IQR)	0.9 (0.4–2.3)	0.9 (0.4–2.2)	1.1 (0.5–3.0)	<0.001
Release site type, n (%)				<0.001
Jail	9330 (70.2)	6914 (66.0)	2416 (86.0)	
Prison	3953 (29.8)	3559 (34.0)	394 (14.0)	
Calendar year of release				<0.001
2017	5175 (39.0)	3731 (35.6)	1444 (51.4)	
2018	6343 (47.8)	5209 (49.7)	1134 (40.4)	
2019	1765 (13.3)	1533 (14.6)	232 (8.3)	

IQR = interquartile range

^a^Percentages may not sum to 100 due to rounding

Compared with those enrolled in Medicaid prior to release, those enrolled post-release were less likely to be female (11.5% versus 18.5%, p<0.001), had a longer median years incarcerated (1.1 [IQR: 0.5–3.0] versus 0.9 [IQR:0.4–2.2], p<0.001), and were more likely to be released from a jail (86.0% versus 66.0%, p<0.001). There were no significant differences in age and education level between the groups.

Among the study population, approximately two thirds (65.6%, n = 8713) had a primary care visit, 57.9% (n = 7693) had an emergency department visit, and 16.3% (n = 2166) had a hospitalization within a year after release (Table 2). For specialty behavioral health services, 14.6% (n = 1935) of the study population received an outpatient mental health service and 13.4% (n = 1179) received a psychiatry service. The most commonly prescribed drugs were antihypertensives (22.1%, n = 2929) and antidepressants (22.0%, n = 2926).

**Table 2:** Medical and specialty behavioral health service use among Louisiana Medicaid members released from state custody between January 1, 2017 and June 30, 2019, within one year after release.

Health service	Overall study population (n = 13283), n (%)	Enrolled in Medicaid pre-release (n = 10473), n (%)	Enrolled in Medicaid post-release (n = 2810), n (%)	P-value
Primary care visit	8713 (65.6)	6782 (64.8)	1931 (68.7)	<0.001
Emergency department visit	7693 (57.9)	6018 (57.5)	1675 (59.6)	0.04
Inpatient hospitalization	2166 (16.3)	1663 (15.9)	503 (17.9)	0.01
Cancer screening				
Cervical cancer^a^	423 (18.9)	363 (19.0)	60 (18.9)	0.99
Colon cancer^b^	281 (13.4)	227 (13.6)	54 (12.9)	0.71
Breast cancer^c^	53 (23.9)	*^d^	*	0.96
Prescription drugs				
Antihypertensive	2929 (22.1)	2393 (22.9)	536 (19.1)	<0.001
Insulin and hypoglycemics	641 (4.8)	540 (5.2)	101 (3.6)	0.001
Inhaled bronchodilator or corticosteroid	1133 (8.5)	945 (9.0)	188 (6.7)	<0.001
HIV antiretroviral	288 (2.2)	258 (2.5)	30 (1.1)	<0.001
Contraceptive^e^	202 (10.9)	185 (11.6)	17 (6.5)	0.01
Cancer chemotherapy	50 (0.4)	*	*	0.37
Specialty behavioral health services				
Psychiatrist visit	1179 (13.4)	1429 (13.6)	350 (12.5)	0.10
Outpatient mental health service	1935 (14.6)	1589 (15.2)	346 (12.3)	<0.001
Inpatient mental health service	767 (5.8)	610 (5.8)	157 (5.6)	0.63
Outpatient substance use disorder service	766 (5.8)	597 (5.7)	169 (6.0)	0.53
Residential substance use disorder service	920 (6.9)	703 (6.7)	217 (7.7)	0.06
Medication for opioid use disorder	773 (5.8)	622 (5.9)	151 (5.4)	0.26
Behavioral health prescription drugs				
Antidepressant	2926 (22.0)	2426 (23.2)	500 (17.8)	<0.001
Antipsychotic	1571 (11.8)	1306 (12.5)	265 (9.4)	<0.001
Mood stabilizer	529 (4.0)	453 (4.3)	76 (2.7)	<0.001

^a^The denominator for this service is women age 21 to 64 years old in the study population (n = 2233)

^b^The denominator for this service is individuals age 50 years and older in the study population (n = 2091)

^c^The denominator for this service is women age 50 and older in the study population (n = 222)

^d^An asterisk indicates that the value was censored due to small cell size

^e^The denominator for this service is women age 44 or younger in the study population (n = 1852)

Compared with those enrolled in Medicaid prior to release, those enrolled post-release were more likely to have a primary care visit (68.7% versus 64.8%, p<0.001), emergency department visit (59.6% versus 57.5%, p = 0.04), and hospitalization (17.9% versus 15.9%, p = 0.01). For cancer screenings, there were no significant differences between the groups. For specialty behavioral health services, compared with those enrolled in Medicaid prior to release, those enrolled post-release were less likely to receive outpatient mental health services (12.3% versus 15.2%, p<0.001), with no significant differences in other services. Compared with those enrolled in Medicaid prior to release, those enrolled post-release were significantly less likely to receive all prescription drugs, except for cancer chemotherapy where there was no significant difference between the two groups.

Of those receiving services, compared with those enrolled in Medicaid prior to release, those enrolled post-release had a significantly longer time to a primary care visit (adjusted mean difference: 42.2 days [95% CI: 37.9 to 46.5; p<0.001]), emergency department visit (adjusted mean difference: 35.8 days [95% CI: 30.1 to 41.6; p<0.001]), and hospitalization (adjusted mean difference: 10.3 days [95% CI: 0.7 to 20.0; p = 0.04], Table 3). For cancer screening, compared with those enrolled in Medicaid prior to release, those enrolled post-release received cervical cancer screening a mean of 39.4 (95% CI: 5.7 to 73.1; p = 0.02) days later. For specialty behavioral health services, compared with those enrolled in Medicaid prior to release, those enrolled post-release received a psychiatrist visit a mean of 22.1 (95% CI: 11.7 to 32.5; p<0.001) days later, outpatient mental health services a mean of 42.8 (95% CI: 31.3 to 54.4; p<0.001) days later, outpatient substance use disorder services a mean of 20.6 (95% CI: 2.0 to 39.2; p = 0.03) days later, and medication for opioid use disorder a mean of 40.4 (95% CI: 23.7 to 57.1; p<0.001) days later. For prescription drugs, compared with those enrolled in Medicaid prior to release, those enrolled post-release received all drugs significantly later, except for cancer chemotherapy. The longest adjusted mean difference was found for contraceptives (68.5 days [95% CI: 32.8 to 104.2, p<0.001]), inhaled bronchodilators and corticosteroids (63.8 days [95% CI: 49.3 to 78.3, p<0.001]), antipsychotics (62.9 days [95% CI: 50.8 to 75.1; p<0.001]), antihypertensives (60.5 days [95% CI: 50.7 to 70.3; p<0.001]), and antidepressants (52.3 days [95% CI: 44.1 to 60.5; p<0.001]).

**Table 3:** Days between release and receipt of medical and specialty behavioral health services among Louisiana Medicaid members released from state custody between January 1, 2017 and June 30, 2019.

Health service	Overall study population (n = 13283), days		Enrolled in Medicaid pre-release (n = 10473), days		Enrolled in Medicaid post-release (n = 2810), days		Adjusted mean difference, days (95% CI)^a^	*P* value
	Mean (SD)	Median (IQR)	Mean (SD)	Median (IQR)	Mean (SD)	Median (IQR)		
Primary care visit	105.4 (91.2)	79 (30–160)	95.6 (91.1)	63 (24–143)	139.9 (83.0)	128 (73 to 192)	42.2 (37.9 to 46.5)	<0.001
Emergency department visit	124.8 (98.9)	101 (41–191)	117.1 (99.3)	90 (32–184)	152.2 (92.5)	140 (75 to 211)	35.8 (30.1 to 41.6)	<0.001
Inpatient hospitalization	153.4 (104.3)	139 (64–236)	151.7 (108.8)	134 (56–243)	159.1 (87.9)	148 (92 to 215)	10.3 (0.7 to 20.0)	0.04
Cancer screening								
Cervical cancer	151.8 (100.9)	133 (62–229)	147.0 (102.7)	126 (56–227)	181.1 (84.2)	183.5 (109.5–233.5)	39.4 (5.7 to 73.1)	0.02
Colon cancer	161.0 (98.3)	146 (82–235)	156.9 (96.6)	141 (79–224)	178.3 (104.2)	154 (96–286)	16.7 (-14.5 to 47.8)	0.30
Breast cancer	178.0 (106.3)	176 (90–261)	170.0 (107.8)	165 (86–256)	222.8 (90.3)	215.5 (148.5–299)	51.1 (-41.7 to 143.9)	0.28
Prescription drugs								
Antihypertensives	109.4 (98.5)	75 (28–172)	97.6 (95.8)	57 (23–149)	161.9 (93.1)	146.5 (81.5–234.5)	60.5 (50.7 to 70.3)	<0.001
Insulin and hypoglycemics	86.9 (89.8)	44 (19–134)	78.1 (87.7)	37 (17–111)	134.2 (86.4)	109 (63–195)	42.4 (24.1 to 60.7)	<0.001
Inhaled bronchodilators and corticosteroids	141.7 (104.8)	116 (48–221)	130.8 (103.2)	100 (41–208)	196.4 (95.9)	201.5 (115–268)	63.8 (49.3 to 78.3)	<0.001
HIV antiretrovirals	85.8 (90.8)	45.5 (22–116.5)	80.4 (88.4)	40 (21–103)	132.5 (99.3)	105.5 (53–233)	35.3 (6.0 to 64.7)	0.02
Contraceptives	142.0 (112.2)	112 (44.5–239.5)	136.2 (112.0)	92 (40–232)	206.1 (96.0)	210 (145–296)	68.5 (32.8 to 104.2)	<0.001
Cancer chemotherapy	126.8 (101.9)	95 (45–193)	128.3 (105.3)	97 (45–203)	119.0 (87.2)	88.5 (60.5–163)	-18.8 (-79.3 to 41.7)	0.54
Specialty behavioral health services								
Psychiatrist visit	145.6 (103.2)	127 (56–223)	141.5 (106.3)	118 (49–225)	162.0 (87.2)	153 (91–223)	22.1 (11.7 to 32.5)	<0.001
Outpatient mental health service	127.8 (102.7)	102 (35–203)	119.7 (102.4)	87 (31–195)	165.1 (95.5)	157 (90–232)	42.8 (31.3 to 54.4)	<0.001
Inpatient mental health service	161.9 (105.2)	152 (69–250)	158.7 (109.3)	146 (61–255)	174.5 (86.7)	160 (122–228)	16.2 (-0.7 to 33.1)	0.06
Outpatient substance use disorder service	151.6 (103.2)	136.5 (58–234)	147.8 (104.1)	127 (55–231)	164.9 (99.2)	154 (79–247)	20.6 (2.0 to 39.2)	0.03
Residential substance use disorder service	173.0 (102.3)	164 (88–256.5)	170.8 (104.6)	162 (84–257)	180.1 (94.3)	176 (100–254)	10.0 (-5.2 to 25.3)	0.20
Medication for opioid use disorder	157.1 (101.5)	143 (67–238)	148.9 (102.9)	133 (58–235)	191.1 (87.8)	195 (119–256)	40.4 (23.7 to 57.1)	<0.001
Behavioral health prescription drugs								
Antidepressants	128.6 (101.3)	103 (39–206)	118.7 (101.2)	86 (33–192)	176.6 (87.5)	171 (105.5–241)	52.3 (44.1 to 60.5)	<0.001
Antipsychotics	137.9 (103.2)	115 (47–218)	127.4 (102.3)	98 (38–201)	189.7 (91.1)	184 (113–262)	62.9 (50.8 to 75.1)	<0.001
Mood stabilizers	145.1 (107.8)	123 (45–229)	139.8 (109.8)	112 (41–226)	176.4 (89.6)	176.5 (99.5–236.5)	27.8 (3.5 to 52.2)	0.03

SD = standard deviation; IQR = interquartile range

^a^Refers to the mean difference in days between release and use of a given service associated with Medicaid enrollment after release, compared with Medicaid enrollment pre-release, after adjusting for sex, length of incarceration, release from jail or prison, and calendar year of release. For example, a positive value indicates that Medicaid enrollment after release was associated with a higher number of days between release and use of a given service, as compared with pre-release Medicaid enrollment.

## Discussion

In a retrospective cohort study of Louisiana Medicaid members released from state custody, we found a significant time gap between release and receipt of many important health services. Further, when compared with Medicaid enrollment prior to release, we found that Medicaid enrollment post-release was associated with a longer time to receipt of virtually all health services, even after adjusting for several differences between the groups.

Overall, we identified evidence of significant gaps in continuity of medical and behavioral health care after release. Time gaps in accessing behavioral health services are especially concerning, given the high proportion of the study population with a substance use disorder and findings from prior research, which indicate that the post-release period is associated with an increased risk of death from overdose [2–4]. Facilitating faster access to behavioral health services is also critical because rates of suicide are higher among people who have been incarcerated than the general population [30].

Further, the mean time between release from state custody and filling of different classes of prescription drugs was often 100 days or more overall. A recent Louisiana legislative report indicated that up to 90 percent of people at some state-operated prisons take prescription medication [23], suggesting that the time gaps we found largely reflect gaps in continuity of treatment of chronic conditions, as opposed to newly diagnosed conditions after release. One explanation of this finding is that policies around medication delivery are inconsistent in Louisiana prisons [23] and people leaving incarceration have reported leaving with no medication at all [20]. Specifically, the documented DPSC practice of hiring physicians with restricted medical licenses, often due to physicians’ personal wrongdoing, makes it impossible for formerly incarcerated people to fill prescriptions written by these providers upon return to the community [31]. Ensuring that only physicians with unrestricted medical licenses can practice in prisons and enrolling them as Medicaid providers would have significant potential to improve continuity of prescription drug treatment.

With respect to Medicaid enrollment, we found that those enrolled in Medicaid prior to release were more likely than those enrolled post-release to receive most medical and behavioral health care services analyzed. Unexpectedly, we found that those enrolled in Medicaid prior to release were significantly less likely to receive a primary care visit; however, the magnitude of this difference was modest. This finding may be due to individuals with specialized medical needs being linked directly with specialists, for example by managed care organization case management programs. Such individuals may include those who receive primary care from a specialist physician (e.g., an infectious diseases specialist for someone with HIV or a nephrologist for someone with end stage renal disease) or individuals with a serious active medical (e.g., cancer) or psychiatric illness (e.g., schizophrenia) where primary care needs are comparatively less urgent. Given that those enrolled in Medicaid prior to release also had lower use of emergency department and inpatient hospital care, the significance of lower primary care use is unclear but may not have adversely affected other outcomes.

Among those receiving services, we found evidence that enrollment prior to release may help reduce time between release and receipt of services. Compared with those enrolled in Medicaid after release, those enrolled pre-release had significantly shorter times to receipt of most health services and all but one prescription medication. The lower time to receipt of prescription drugs may explain, at least in part, why those enrolled in Medicaid prior to release had significantly lower use of emergency department and inpatient hospital care than those enrolled post-release. Although time to receipt of care was high regardless of when a person was enrolled, these findings point to the value of pre-release Medicaid enrollment efforts in improving continuity of care and mitigating delays.

Our findings add to existing data showing the need for additional support for individuals released from state custody to engage in health care. Broadly, 65.6% of people in the study population used primary care services versus 75.5% of the general adult Louisiana Medicaid population in 2020 [32]. Health care in Louisiana prisons is provided on a “sick call” system (i.e. patients request health services when ill or injured) and regular preventive health care visits for people under 50 years of age are not offered [23]. Many recently released people may have gone years without appropriate preventive care due to a myriad of documented barriers to receiving care including medical fees that are disproportionate to incarcerated wages, lack of appropriately trained providers, distrust of providers, and having health concerns ignored [20–23]. Lack of prevention and engagement in primary care during incarceration may explain why over half of the study population had an emergency department visit, as opposed to just 37% of Medicaid members nationally in 2018 [33]. Finally, low rates of cervical and breast cancer screening among the study population, combined with qualitative evidence that these services are not consistently available during incarceration [29] suggest that after release, women may experience a gap in care.

One option for improving care transitions—both in Louisiana and elsewhere—would be expanding evidence-based models such as the Transitions Clinic Network, which relies on formerly incarcerated community health workers to engage people in primary care during the transitional period [34]. While community health worker services have historically been grant-funded, several states now have Medicaid State Plan Amendments that allow for reimbursement for some of their services, including in health system navigation [35]. In addition, 41 states (including DC) contract with Medicaid managed care organizations to provide coverage for Medicaid members. States are increasingly requiring that their contracted Medicaid managed care organizations cover community health worker services [36] and could add stipulations that these workers serve formerly incarcerated Medicaid members, particularly those identified as having substantial medical or behavioral health needs.

The Centers for Medicare and Medicaid Services (CMS) recently approved California’s Reentry Demonstration Initiative will allow Medicaid to cover some services for incarcerated people (e.g. substance use treatment, case management, and community health worker services) for 90 days prior to release, with the goal of improving care coordination and preventing suicide, overdose, and recidivism. As of February 2023, an additional 14 states have also requested some waiver of the Federal Inmate Exclusion Policy. Evaluation of approved waivers will have tremendous implications for other states [37].

This study has several limitations. First, results may not be generalizable to other states or other time periods. Second, we used social security numbers to link Medicaid and Department of Public Safety and Corrections release data and so data entry errors may have led to mismatches or failure to match for some otherwise eligible individuals. Third, race/ethnicity information in Medicaid claims was limited, resulting in a high proportion of individuals with a recorded race/ethnicity value of missing or unknown. Of note, 32.5% of the state prison population is white and 67.1% Black, as of December 2020 according to the Department of Public Safety and Corrections [19]. Fourth, release from prison versus jail was determined by analyzing facility codes manually which may have resulted in misclassification. Fifth, estimates of the percentage of eligible individuals who received cancer screening may be underestimates due to screening tests that may have occurred during or prior to incarceration; however, previous work has found that cancer screening in jails and prisons is limited [38–41]. Finally, it is possible that some people who enrolled in Medicaid post-release were generally healthier than those who enrolled earlier, and thus needed fewer medications and health services.

Overall, Medicaid enrollment prior to release is associated with faster access to a wide variety of health services among Medicaid members released from state custody. However, even with Medicaid enrollment prior to release, we found prolonged times between release and receipt of services, particularly behavioral health services and prescriptions. States should consider implementing programs to enroll people in Medicaid prior to release from incarceration and make efforts to improve care coordination for people transitioning out of incarceration.

## Supporting information

S1 Checklist STROBE statement—checklist of items that should be included in reports of observational studies. (DOCX)
