# Supplementary material for: Health services use among formerly incarcerated Louisiana Medicaid members within one year of release
Source: PLoS One. 2023 May 18;18(5):e0285582. doi: 10.1371/journal.pone.0285582 (PMC10194928; doi:10.1371/journal.pone.0285582)
Supplement: S1 Checklist — (DOCX) [file pone.0285582.s001.docx]

STROBE Statement—checklist of items that should be included in reports of observational studies

|  | Item No. | Recommendation | Page  No. | Relevant text from manuscript |
| --- | --- | --- | --- | --- |
| **Title and abstract** | 1 | (*a*) Indicate the study’s design with a commonly used term in the title or the abstract | 2 | “We conducted a retrospective cohort study…” |
|  |  | (*b*) Provide in the abstract an informative and balanced summary of what was done and what was found | 2-3 | Abstract, Methods and Results |
| Introduction | | | |  |
| Background/rationale | 2 | Explain the scientific background and rationale for the investigation being reported | 5-7 | Introduction section |
| Objectives | 3 | State specific objectives, including any prespecified hypotheses | 7 | “The aims were to…” |
| Methods | | | |  |
| Study design | 4 | Present key elements of study design early in the paper | 8 | Methods, Overview subsection |
| Setting | 5 | Describe the setting, locations, and relevant dates, including periods of recruitment, exposure, follow-up, and data collection | 8-10 | Methods section |
| Participants | 6 | (*a*) *Cohort study*—Give the eligibility criteria, and the sources and methods of selection of participants. Describe methods of follow-up  *Case-control study*—Give the eligibility criteria, and the sources and methods of case ascertainment and control selection. Give the rationale for the choice of cases and controls  *Cross-sectional study*—Give the eligibility criteria, and the sources and methods of selection of participants | 8-9 | Methods, Study population subsection |
|  |  | (*b*) *Cohort study*—For matched studies, give matching criteria and number of exposed and unexposed  *Case-control study*—For matched studies, give matching criteria and the number of controls per case | N/A, no matching |  |
| Variables | 7 | Clearly define all outcomes, exposures, predictors, potential confounders, and effect modifiers. Give diagnostic criteria, if applicable | 9-10 | Methods, Health conditions and service use, Medicaid enrollment, and Other characteristics subsections |
| Data sources/ measurement | 8* | For each variable of interest, give sources of data and details of methods of assessment (measurement). Describe comparability of assessment methods if there is more than one group | 9-10 | Methods, Health conditions and service use, Medicaid enrollment, and Other characteristics subsections |
| Bias | 9 | Describe any efforts to address potential sources of bias | 11 | Methods, Health conditions and service use subsection |
| Study size | 10 | Explain how the study size was arrived at | 8 | Methods, Overview subsection |

Continued on next page

| Quantitative variables | 11 | Explain how quantitative variables were handled in the analyses. If applicable, describe which groupings were chosen and why | 9-10 | Methods, Health conditions and service use, Medicaid enrollment, and Other characteristics subsections |
| --- | --- | --- | --- | --- |
| Statistical methods | 12 | (*a*) Describe all statistical methods, including those used to control for confounding | 11 | Methods, Statistical analysis subsection |
|  |  | (*b*) Describe any methods used to examine subgroups and interactions | N/A – no subgroups analyzed in this study |  |
|  |  | (*c*) Explain how missing data were addressed | 11 | “First, using a complete case analysis…” |
|  |  | (*d*) *Cohort study*—If applicable, explain how loss to follow-up was addressed  *Case-control study*—If applicable, explain how matching of cases and controls was addressed  *Cross-sectional study*—If applicable, describe analytical methods taking account of sampling strategy | N/A |  |
|  |  | (*e*) Describe any sensitivity analyses | N/A |  |
| Results | | | | |
| Participants | 13* | (a) Report numbers of individuals at each stage of study—eg numbers potentially eligible, examined for eligibility, confirmed eligible, included in the study, completing follow-up, and analysed | 11 | “Of 13287 individuals meeting study criteria, complete data were available for 13283.” |
|  |  | (b) Give reasons for non-participation at each stage | 11 | See 13a |
|  |  | (c) Consider use of a flow diagram | N/A | Reporting via manuscript text is straightforward |
| Descriptive data | 14* | (a) Give characteristics of study participants (eg demographic, clinical, social) and information on exposures and potential confounders | Table 1 |  |
|  |  | (b) Indicate number of participants with missing data for each variable of interest | Table 1 |  |
|  |  | (c) *Cohort study*—Summarise follow-up time (eg, average and total amount) | N/A, follow up was 1 year uniformly | Methods, Health conditions and service use subsection |
| Outcome data | 15* | *Cohort study*—Report numbers of outcome events or summary measures over time | Table 2 and 3 |  |
|  |  | *Case-control study—*Report numbers in each exposure category, or summary measures of exposure |  |  |
|  |  | *Cross-sectional study—*Report numbers of outcome events or summary measures |  |  |
| Main results | 16 | (*a*) Give unadjusted estimates and, if applicable, confounder-adjusted estimates and their precision (eg, 95% confidence interval). Make clear which confounders were adjusted for and why they were included | Table 2 and 3 |  |
|  |  | (*b*) Report category boundaries when continuous variables were categorized | N/A |  |
|  |  | (*c*) If relevant, consider translating estimates of relative risk into absolute risk for a meaningful time period | Table 3 |  |

Continued on next page

| Other analyses | 17 | Report other analyses done—eg analyses of subgroups and interactions, and sensitivity analyses | N/A |  |
| --- | --- | --- | --- | --- |
| Discussion | | | | |
| Key results | 18 | Summarise key results with reference to study objectives | 17-21 | Discussion section |
| Limitations | 19 | Discuss limitations of the study, taking into account sources of potential bias or imprecision. Discuss both direction and magnitude of any potential bias | 21-22 | Discussion, Limitations subsection |
| Interpretation | 20 | Give a cautious overall interpretation of results considering objectives, limitations, multiplicity of analyses, results from similar studies, and other relevant evidence | 17-22 | Discussion section |
| Generalisability | 21 | Discuss the generalisability (external validity) of the study results | 21 | Discussion, Limitations subsection |
| Other information | |  | | |
| Funding | 22 | Give the source of funding and the role of the funders for the present study and, if applicable, for the original study on which the present article is based | Funding statement |  |

*Give information separately for cases and controls in case-control studies and, if applicable, for exposed and unexposed groups in cohort and cross-sectional studies.

**Note:** An Explanation and Elaboration article discusses each checklist item and gives methodological background and published examples of transparent reporting. The STROBE checklist is best used in conjunction with this article (freely available on the Web sites of PLoS Medicine at http://www.plosmedicine.org/, Annals of Internal Medicine at http://www.annals.org/, and Epidemiology at http://www.epidem.com/). Information on the STROBE Initiative is available at www.strobe-statement.org.
